# Incidence of neuromyelitis optica spectrum disorder (NMOSD) in China: A national population-based study

**DOI:** 10.1016/j.lanwpc.2020.100021

**Published:** 2020-09-06

**Authors:** De-Cai Tian, Zixiao Li, Meng Yuan, Chengyi Zhang, Hongqiu Gu, Yongjun Wang, Fu-Dong Shi

**Affiliations:** a China National Clinical Research Center for Neurological Diseases, Beijing Tiantan Hospital, Capital Medical University, Beijing 100070, China; b Department of Neurology, Tianjin Neurological Institute, Tianjin Medical University General Hospital, 154 Anshan Rd, Heping District, Tianjin 300052, China

**Keywords:** NMOSD, Incidence, Comorbidity, Mortality

## Abstract

**Background:**

Neuromyelitis Optica Spectrum Disorder (NMOSD) is an inflammatory disease of the central nervous system with preferential involvement of the optic nerve and spinal cord. However, data on NMOSD incidences in China, a country encompassing 20% of the world's population and covering vast areas of Eastern Asia, are unknown.

**Methods:**

We conducted the first nationwide survey of NMOSD, based on the database of the National Hospital Quality Monitoring System (HQMS) of China, which covers all 1665 tertiary hospitals. The “Medical Record Homepage” of all patients were consistently collected via a standard protocol across each tertiary hospital. The primary outcome was the incidence of NMOSD, diagnosed according to the 2015 International Panel for Neuromyelitis Optica Diagnosis criteria and identified by ICD-10 code (G36•0). Burden of hospitalization, comorbidities, and death were also evaluated.

**Findings:**

We identified 33,489 hospital admissions for 17,416 NMOSD diagnosed patients from 2016 to 2018. 11,973 patients were newly diagnosed NMOSD. The age and sex adjusted incidence per 100,000 person years was 0•278 (95% confidence interval [CI], 0•273–0•283), with 0•075 (0•069–0•08) in child and 0•347 (0•34–0•353) in adult. The peak age of onset NMOSD is 45–65 years with an incidence 0•445/100,000 (95% CI, 0•433–0•457). The female to male ratio was 4•71 (*p*<0•001, 95% CI, 4•50–4•94). Geographical distribution of NMOSD is not related to the latitude gradient. Sjögren's syndrome (1,124/17,416, 6•5%) and systemic lupus erythematosus (387/17,416, 2•2%) are the most frequently autoimmune comorbidities. 106 adults and 4 children of the 17,416 NMOSD cohort died.

**Interpretation:**

The incidence of NMOSD in China per 100,000 person years was 0•278, with 0•075 in child and 0•347 in adult. The geographical distribution of NMOSD is not related to the latitude gradient.

**Funding:**

National Science Foundation of China (91949208, 91642205, and 81830038); Advanced Innovation Center for Human Brain Protection, Capital Medical University, Beijing.

**Research in context**

**Evidence before this study**

A systematic search of PubMed and the web of science was conducted from 1985 up to 2020, with theme subject defined as (" neuromyelitis optica " OR " neuromyelitis optica spectrum disorder ") AND (prevalence OR incidence OR epidemiology) AND (China OR Chinese OR "Hong Kong" OR Taiwan). Resultingly, there are 2, 1, and 0 related articles in Taiwan, Hong Kong, and Mainland China, respectively. From these regional studies, we extrapolated that the average incidence of pediatric neuromyelitis optica (NMO) during 2011–2015 was 0.11 per 100,000 person-years in Taiwan. Neuromyelitis optica spectrum disorder (NMOSD) accounts for 22•7% of inflammatory demyelinating disorders among Hong Kong Chinese from 1980 to 2010 in a hospital-based study. Collectively, epidemiological studies on NMOSD are scarce in China.

**Added value of this study**

This is the first-ever nation-wide survey of NMOSD in mainland China. We estimate the age- and sex-adjusted incidence of NMOSD across all age groups at is 0•278 per 100,000 persons, with 0•075 in child and 0•347 in adult. Meanwhile, a map of the incidence rates in 31 provinces and municipalities is compiled in mainland China. The peak age of onset NMOSD is 45–65 years and the female to male ratio is 4•71. As the latest government effort to reform China's health care system, the Urban Resident Basic Medical Insurance and New Rural Cooperative Medical Insurance cover about 68% of all patients, and the hospitalization burden display a downward trend. Comorbidities and mortality of NMOSD are also evaluated.

**Implication of all the available evidence**

The NMOSD incidence rate in China is 0•278 per 100,000 person-years, which is comparable with East Asian countries. For the first time, our study reveals the epidemiologic features of NMOSD in China, these results enrich the global map of NMOSD. This national administrative database enhances the accuracy of estimates for policy makers, providers to improve health-service planning. The reported disease burden calls for dramatically increased regional and global efforts in NMOSD patient care as well as investment in research for this devastating disease.

Alt-text: Unlabelled box

## Introduction

Neuromyelitis optica spectrum disorder (NMOSD) is a severe inflammatory disease of the central nerves system (CNS) with preferential involvement of optic nerve and spinal cord [1]. The discovery of highly specific auto-antibodies against aquaporin-4 (AQP4) in 2005 has characterized NMOSD as new disease entity distinct from multiple sclerosis (MS) [2, 3] Although NMOSD occurs all over the world, a predilection for non-Caucasians has been proposed, especially observed in Asian countries [4]. The ratio of NMOSD to MS is higher in Asian than in Western countries [5].

Amongst Asian countries, the prevalence of NMOSD was 3•42/100,000 in Japan [6], 2•56/100,000 in Korea [7], and 1•94/100,000 in Malaysia [8]. In contrast, population-based studies of NMOSD indicated that the prevalence was 0•70/100, 000 in Australia and New Zealand [9], 1•04/100,000 in Sweden [10], and 1•09 per 100,000 in Denmark [11]. The worldwide variation of NMOSD incidence ranges from 0.037/100,000 in Australia and New Zealand to 0.73/100,000 person-years in Korea and Martinique [7, 9, 12]. Asian populations are three times more likely to develop NMOSD (1•57 VS. 0•57 per 100,000) compared with the Caucasian populations in Australia and New Zealand [9].

Although these latest findings have emerged, the gaps in epidemiological studies of NMOSD remain conspicuous. As the world's most populous country, China with an estimated population of 1•4 billion represents 20% of all people on Earth. Since the first case of MS was described in Peking Union Hospital a century ago, there still lacks a nationwide survey for the prevalence and incidence of neuroinflammatory diseases [13]. Presently, most Chinese studies on MS and NMOSD based on case series. There is no study on incidence of NMOSD in China. Therefore, we aim to estimate the incidence of NMOSD based on a nationwide mandatory database of Hospital Quality Monitoring System (HQMS), which is maintained by National Health Commission (NHC) in China [14].

## Methods

### Data sources and collection

This nationwide study is based on a national administrative database covering the entire Chinese population. The People's Republic of China (mainland China), located in East Asia (latitudes between 18°24 N and 52°33 N) with a population of 1•39538 billion in 2018. Although China is a large multi-ethnic state, 91•51% of Chinese population is Han Chinese and the other 55 minority ethnic groups are distributed extensively throughout diverse and localized regions. The Hospital Quality Monitoring System (HQMS), launched in 2011, is an official data collection system covering all tertiary hospitals [14, 15]. As part of China's health system reform, the HQMS is designed to monitor the quality of medical care as well as provide metrics for performance appraisal of all tertiary public hospitals. The database is designed to link hospital information systems and automatically compile the inpatient medical record of every public tertiary hospital. The data quality and consistency are enforced through a unified transmission protocol (appendix section 2). The database collects a “Medical Record Homepage”, a summary of the patient's hospitalization information, including 346 variables such as demographic characteristics, diagnoses, procedures, and expenses etc. Given the vast geographical boundaries and large population mobility of China, this unique nationwide patient identification system allows linkage across temporal and geographic barriers.

### Study population

We retrieved 240,401 hospitalization records from the HQMS database between 1st, January 2016 to 31st, December 2018 based on the diagnosis of inflammatory demyelinating disease (IDD). This study includes a total of 1665 tertiary hospitals in 31 provinces and municipalities throughout mainland China and excludes special administrative zones of Hong Kong and Macau. According to the China Health Statistics Yearbook 2018, there are 2340 tertiary hospitals in China. The 1665 hospitals surveyed covered 98•5% of tertiary public western medicine hospitals, where NMOSD patients are diagnosed and managed. The rest 228 private hospitals and 422 Chinese medicine hospitals do not accommodate these patients. Appendix Figure 1 displays the distribution of the 1665 hospitals across mainland China. All hospitals with a resident neuroimmunological diseases specialist are included (Appendix Table 1). Generally, NMOSD patients were admitted for inpatient care due to vision loss or paralysis; in China the majority of these patients with IDD are referred to public tertiary hospitals. Patients in the HQMS database are referred from a spectrum of departments, such as neurology, emergency, ophthalmology, pediatrics and rehabilitation, thus, ensuring maximum coverage for NMOSD patients. The study population (denominator) included the entire population, from all ages throughout 2016 to 2018, according to annual reports of the National Bureau of Statistics of China which provides precise and in-depth information on China's mainland population.

### Case ascertainment

The HQMS was queried for all discharges from all departments with IDD as a primary diagnosis, or as an auxiliary diagnosis throughout the 2016–2018 period. As a group of heterogeneous autoimmune inflammatory diseases, IDD of CNS includes MS, NMOSD, acute disseminated encephalomyelitis (ADEM), optic neuritis (ON), acute myelitis (AM), and unclassified IDD (uIDD) [16]. These diseases have corresponding International Classification of Diseases, 10th revision (ICD-10) code (see appendix section 2). Patient identification code, demographic details (age, sex, native province and ethnicity), diagnosis, hospital costs, payment methods, comorbidities, and causes of death, were all collected.

**Fig. 1:**
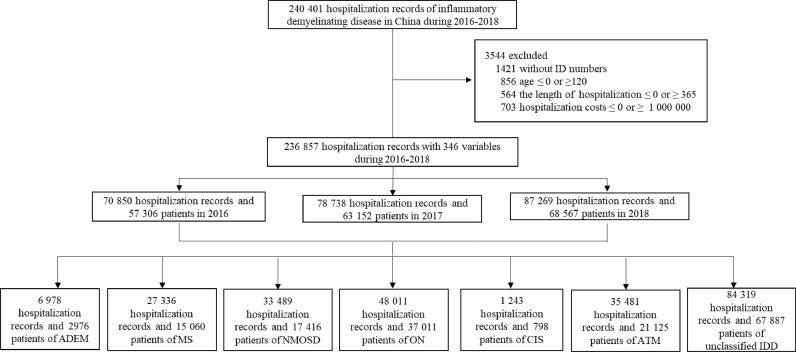
**The incidence of NMOSD in China: flowchart of study population selection.** ADEM: acute disseminated encephalomyelitis; ON: optic neuritis; ATM: acute transverse myelitis; uIDD: unclassified IDD. MS: multiple sclerosis; NMOSD: neuromyelitis optica spectrum disorders; ADEM: acute disseminated encephalomyelitis; ON: optic neuritis; ATM: acute transverse myelitis; uIDD: unclassified IDD.

NMOSD was defined according to International Classification of Diseases, Tenth Revision (ICD-10) code (G36•0), diagnosis is based upon the 2015 International Panel for Neuromyelitis Optica Diagnosis criteria [17]. Cell based assay (CBA) and enzyme linked immunosorbent assay (ELISA) are the two commonly adopted methods for testing AQP-4 antibody. MRI is routinely administered for diagnosis and follow up in all 1665 tertiary hospitals in China [18], [19], [20]. HQMS requires a Quality Assurance Physician and coder for each medical record, the former reviews the diagnosis, and the coder affirms the ICD-10 code. The Quality Assurance Physicians have access to the entire medical records, including medical history, physical examination, laboratory results, and imaging reports. Of 240,401 hospitalization records for IDD, 33,489 records and 17,416 patients of NMOSD were identified from 2016 to 2018 (Fig. 1). Based on the patient's name, sex, and ID number, “the same person” tag is generated within the HQMS database, thereby avoiding double counting and facilitates a whole of population analysis by age, sex, and region. Newly diagnosed cases are defined as patients who are first recorded in the HQMS database during 2011 to 2018 and primarily diagnosed with NMOSD. New patients with MS in each province or municipality were determined based on the "from province" variable. The details of analysis protocol are described in Section 2 of the appendix. The following definitions for adults and children are used to ensure consistency within WHO guideline. An adult is a person older than 19 years of age, and a child is a person 19 years or younger.

This study was performed at the China National Clinical Research Center for Neurological Diseases and China National Center for Quality Control of Neurological Diseases. This study was approved by the Institutional Review Board of the Beijing Tiantan Hospital.

### Statistical analysis

Incidence referred to the number of new confirmed NMOSD cases occurring during the study period between January 1, 2016 to December 31, 2018. The yearly incidence rate was defined as the number of new NMOSD cases divided by the total population in 2016, 2017, and 2018 (http://www.stats.gov.cn/). The crude incidence with 95% Confidence Interval (CI) of the provinces and municipalities was calculated per 100,000 person years based on a Poisson distribution. The national incidence was estimated by adjusting for sex and age distribution using 2010 national census. Data were presented as mean and 95% CI or median with range (25th and 75th percentiles) for the number of inpatient hospitalizations, number of days in the hospital, and cost of each encounter. *P*< .05 is considered statistically significant. Statistical analyses were performed using SAS version 9•4 (SAS Institute Inc., Cary, NC, USA).

### Role of the funding source

The funding sources of this study did not influence nor participate in study design, data collection, data analysis, data interpretation, or drafting of the report. The corresponding author had full access to all the data in the study as well as final responsibility for the decision to submit for publication.

## Results

### Incidence of NMOSD in China

**Fig. 2:**
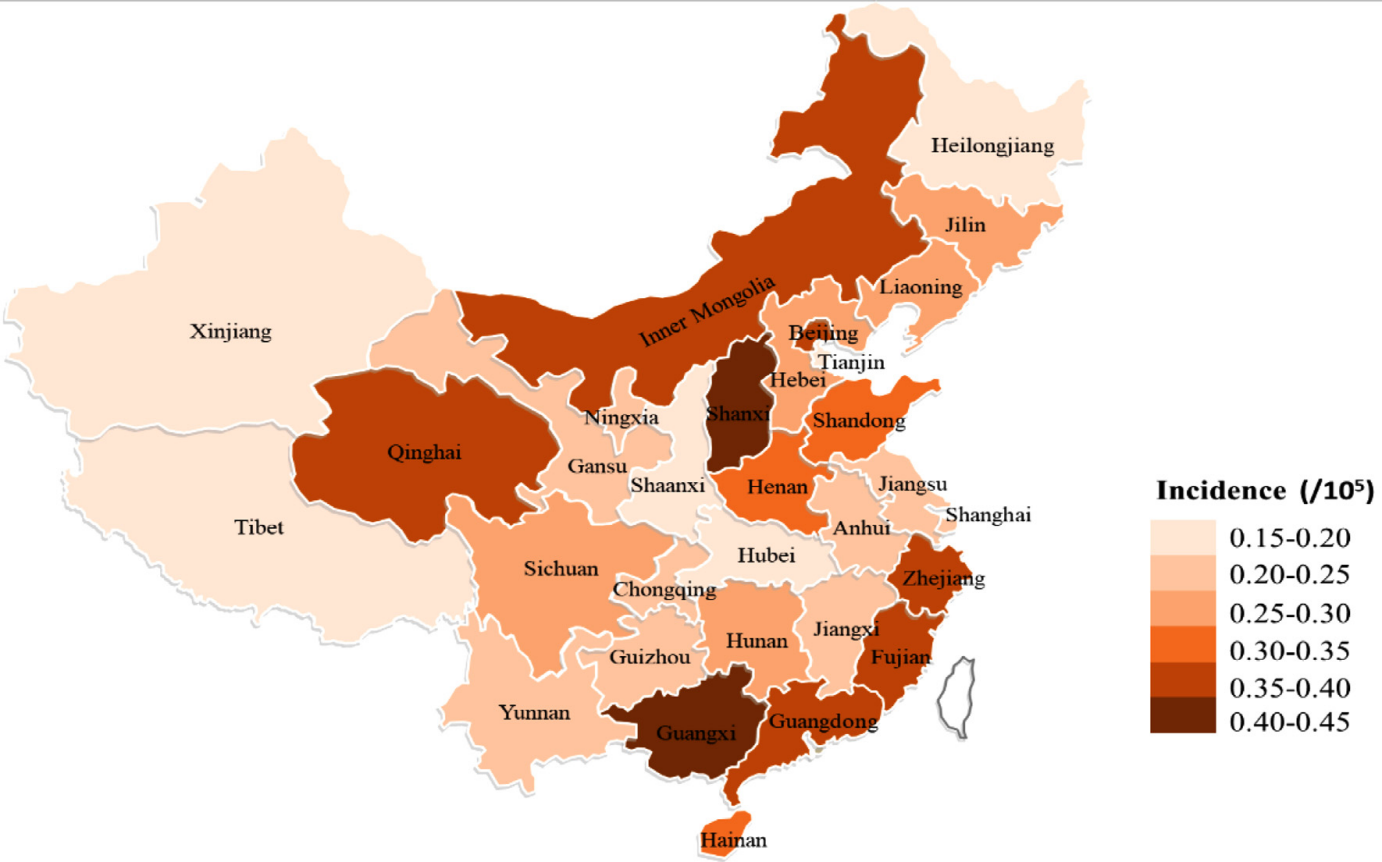
**Incidence map of NMOSD in China, 2016–2018.** The study was conducted in mainland China. Hong Kong and Macao were not included.

We identified 33,489 hospital admissions for 17,416 patients of NMOSD from 2016 to 2018. Amongst these patients, 11,973 people with NMOSD were newly diagnosed: 3796 in 2016, 3923 in 2017, and 4254 in 2018. The overall crude incidence of NMOSD in the Chinese is 0•287 (95% CI, 0•282-0•292) per 100,000 person years during the study period, with 0•074 (0•069–0•08) in children, and 0•347 (0•341–0•354) in adults. The age and sex adjusted incidence per 100,000 person years was 0•278 (95% CI, 0•273–0•283), with 0•075 (0•069–0•08) in children and 0•347 (0•34–0•353) in adults. The incidence of NMOSD in adults is significantly higher than children (*p* < 0•001). Table 1 summarizes the incidence of NMOSD in these different age groups. The peak incidence was among those aged 45–65 years, range from 0•431 (95% CI, 0•41–0•452) to 0•462 (95% CI, 0•44–0•485). A female pre-dominance was observed, where the female to male ratio was 4•71 (95% CI,4•50–4•94, *p*<0•001). The latitude gradient discovered for MS risk was not seen in NMOSD in our study (*B*=−0.002, *p*=0.306) [21]. The estimated crude incidence of NMOSD per 100,000 person-years varied from 0•159 (0•081–0•237) in Tibet (latitude 30° N) and 0•155 (0•132–0•178) in Heilongjiang (latitude 46° N) to 0•416 (0•383–0•449) in Guangxi (latitude 23° N) and 0•425 (0•387–0•463) in Shanxi (latitude 36° N) (Fig. 2).

**Table 1:** Incidence of NMOSD in different age groups in China.

Variables (age group)	2016		2017		2018		Annual incidence rate per 100,000 person-years (95% CI)
	Incident cases (*n*)	Incidence rate per 100,000 person-years (95% CI)	Incident cases (*n*)	Incidence rate per 100,000 person-years (95% CI)	Incident cases (*n*)	Incidence rate per 100,000 person-years (95% CI)	
0–4	9	0•011 (0•004–0•018)	12	0•014 (0•006–0•023)	20	0•024 (0•014–0•035)	0•017 (0•012–0•022)
5–9	33	0•043 (0•029–0•058)	43	0•056 (0•039–0•073)	55	0•071 (0•052–0•09)	0•057 (0•047–0•067)
10–14	38	0•053 (0•036–0•069)	54	0•073 (0•054–0•093)	67	0•088 (0•067–0•109)	0•072 (0•061–0•083)
15–19	112	0•152 (0•124–0•181)	117	0•163 (0•133–0•192)	118	0•166 (0•136–0•196)	0•16 (0•143–0•177)
20–24	195	0•206 (0•177–0•235)	213	0•24 (0•208–0•272)	174	0•21 (0•179–0•241)	0•219 (0•201–0•236)
25–29	331	0•26 (0•232–0•288)	306	0•25 (0•222–0•278)	324	0•286 (0•255–0•317)	0•265 (0•248–0•282)
30–34	301	0•288 (0•255–0•32)	339	0•314 (0•281–0•347)	369	0•325 (0•292–0•358)	0•309 (0•29–0•328)
35–39	325	0•338 (0•301–0•375)	325	0•324 (0•289–0•36)	342	0•343 (0•306–0•379)	0•335 (0•314–0•356)
40–44	424	0•375 (0•339–0•411)	404	0•379 (0•342–0•416)	369	0•362 (0•325–0•399)	0•372 (0•351–0•393)
45–49	536	0•429 (0•393–0•465)	497	0•388 (0•354–0•422)	594	0•476 (0•438–0•514)	0•431 (0•41–0•452)
50–54	527	0•452 (0•414–0•491)	533	0•454 (0•415–0•492)	568	0•481 (0•442–0•521)	0•462 (0•44–0•485)
55–59	316	0•444 (0•395–0•493)	307	0•423 (0•375–0•47)	416	0•489 (0•442–0•536)	0•454 (0•426–0•481)
60–64	327	0•405 (0•361–0•448)	365	0•442 (0•397–0•487)	375	0•452 (0•407–0•498)	0•433 (0•407–0•459)
65–69	173	0•299 (0•254–0•344)	224	0•358 (0•311–0•405)	261	0•391 (0•343–0•438)	0•351 (0•325–0•378)
70–74	84	0•222 (0•175–0•27)	101	0•255 (0•206–0•305)	116	0•273 (0•224–0•323)	0•251 (0•223–0•28)
75–79	52	0•194 (0•141–0•247)	59	0•216 (0•161–0•271)	57	0•205 (0•152–0•258)	0•205 (0•174–0•236)
80–84	11	0•064 (0•026–0•102)	17	0•095 (0•05–0•14)	25	0•138 (0•084–0•192)	0•1 (0•073–0•127)
≥85	2	0•019 (−0•007 to 0•046)	7	0•064 (0•017–0•111)	4	0•035 (0•001–0•069)	0•04 (0•018–0•061)
Male	632	0•09 (0•083–0•097)*	709	0•1 (0•093–0•108)*	807	0•114 (0•106–0•122)*	0•102 (0•097–0•106)*
Female	3164	0•464 (0•448–0•481)*	3214	0•470 (0•454–0•486)*	3347	0•502 (0•485–0•519)*	0•479(0•469–0•488)*
Total	3796	0•268 (0•259–0•276)*	3923	0•275 (0•266–0•284)*	4254	0•292 (0•283–0•301)*	0•278 (0•273–0•283)*

⁎ Adjusted with age and sex.

### Burden of hospitalization

The average length of hospitalization for adult patients with NMOSD decreased from 14•8 ± 11•7 days in 2016 to 14•1 ± 11•6 days in 2018. This trend is similarly observed in children, with the average length of stay reduced from 13•6 ± 9•4 days in 2016 to 12•8 ± 10•6 days in 2018. The average cost of each hospitalization in both, whether adults and or children is decreasing annually. The average cost of each stay decreased from $1975(IQR, 1130-3146) in 2016 to $1822(IQR, 1038–2971) in 2018 in adults. On average, children spend more on hospitalization than adults, but the cost of hospitalization is also lower in 2018 than in 2016 ($2171 versus $2696). As the latest government effort to reform China's health care system, the Urban Employee Basic Medical Insurance (UEBMI), Urban Resident Basic Medical Insurance (URBMI) and the New Rural Cooperative Medical Insurance (NRCMI) dramatically expand people's access to social health insurance. In terms of medical payment methods, UEBMI, URBMI and NRCMI covered the cost of about 68•2% adult patients, 17•6% patients are fully self-funded, and that of which percentage are decreasing per annum. The percentage of children's self-funded accounts is relatively high, at 21•3%. Only 0•34% of NMOSD patients purchased commercial health insurance (Table 2).

**Table 2:** Hospitalization cost, payment methods, and length of stay due to NMOSD.

	2016 (*n*=4580)		2017 (*n*=5768)		2018 (*n*=7068)	
	Child (*n*=262)	Adult (*n*=4318)	Child (*n*=344)	Adult (*n*=5424)	Child (*n*=413)	Adult (*n*=6655)
**Payment methods, No. (%)**						
UEBMI	7 (2•7)	1121 (26)	32 (9•3)	1506 (27•8)	37 (9)	2025 (30•4)
URBMI	72 (27•5)	650 (15•1)	98 (28•5)	914 (16•9)	140 (33•9)	1476 (22•2)
NRCMI	76 (29)	1078 (25)	95 (27•6)	1139 (21)	100 (24•2)	1109 (16•7)
CHI	1 (0•4)	14 (0•3)	1 (0•3)	17 (0•3)	3 (0•7)	24 (0•4)
Fully self-funded	68 (26)	861 (19•9)	63 (18•3)	969 (17•9)	83 (20•1)	1029 (15•5)
Poverty Relief	0 (0)	1 (0)	1 (0•3)	16 (0•3)	2 (0•5)	26 (0•4)
Other Social Insurance	6 (2•3)	96 (2•2)	21 (6•1)	119 (2•2)	12 (2•9)	159 (2•4)
Other	32 (12•2)	497 (11•5)	33 (9•6)	744 (13•7)	36 (8•7)	807 (12•1)
Hospitalization cost, Median (IQR), US$*	2696 (1305–4756)	1975 (1130-3146)	2179 (1044–4181)	1891 (1101–3094)	2171 (1073–4165)	1822 (1038–2971)
Length of hospital stay, Mean (SD), days	13•6 (9•4)	14•8 (11•5)	12•9 (10•7)	14•4 (11•8)	12•8 (10•6)	14•1 (11•6)

UEBMI: The Urban Employee Basic Medical Insurance; URBMI: The Urban Resident Basic Medical Insurance; NRCMI: The New Rural Cooperative Medical Insurance; CHI: Commercial Health Insurance.

⁎ According to the annual exchange rate (average or standardized measure) for the Chinese Yuan to the U.S. dollar.

### Associated comorbidities in patients with NMOSD

Amongst all hospitalized adult patients with NMOSD, the percentage of risk factors for vascular diseases such as hypertension, diabetes, and hyperlipidemia were 14•1%, 7•1%, and 13•4%, respectively. As a result, from 2016 to 2018, the percentage NMOSD patients with combined stroke was 1203/17,416 (7%). Moreover, 608 of 17,416 (3•5%) patients with NMOSD developed osteoporosis. Psychiatric comorbidities were common in NMOSD patients, with about 732/17,416 (4•2%) patients suffering from anxiety and/or depression. Autoimmune conditions were generally high among patients with NMOSD. Among systemic autoimmune diseases, Sjögren's syndrome (1124/17,416, 6•5%) and systemic lupus erythematosus (SLE) (387/17,416, 2•2%) represent the most frequent occurring comorbidities of NMOSD (Table 3).

**Table 3:** Comorbidities of NMOSD during 2016–2018.

Comorbidities, No. (%)	2016 (*n*=4580)		2017 (*n*=5768)		2018 (*n*=7068)	
	Child (*n*=262)	Adult (*n*=4318)	Child (*n*=344)	Adult (*n*=5424)	Child (*n*=413)	Adult (*n*=6655)
Hypertension	4 (1•5)	572 (13•2)	1 (0•3)	750 (13•8)	1 (0•2)	991 (14•9)
Diabetes	1 (0•4)	274 (6•3)	2 (0•6)	374 (6•9)	3 (0•7)	515 (7•7)
Hyperlipidemia	3 (1•1)	551 (12•8)	3 (0•9)	752 (13•9)	4 (1)	891 (13•4)
Osteoporosis	0 (0)	146 (3•4)	0 (0)	204 (3•8)	0 (0)	258 (3•9)
Stroke	1 (0•4)	283 (6•6)	0 (0)	401 (7•4)	1 (0•2)	517 (7•8)
Malignant tumor	0 (0)	37 (0•9)	0 (0)	66 (1•2)	1 (0•2)	79 (1•2)
Depression/anxiety	2 (0•8)	171 (4)	4 (1•2)	220 (4•1)	4 (1)	331 (5)
Autoimmune diseases	15 (5•7)	379 (8•8)	11 (3•2)	586 (10•8)	18 (4•4)	741 (11•1)
Bechet's disease	0 (0)	7 (0•2)	0 (0)	2 (0)	0 (0)	4 (0•1)
Sjögren's syndrome	5 (1•9)	261 (6)	4 (1•2)	372 (6•9)	5 (1•2)	477 (7•2)
SLE	8 (3•1)	78 (1•8)	5 (1•5)	137 (2•5)	11 (2•7)	148 (2•2)
Arthritis	0 (0)	1 (0)	0 (0)	0 (0)	0 (0)	1 (0)
Other	2 (0•8)	32 (0•7)	2 (0•6)	75 (1•4)	2 (0•5)	111 (1•7)

SLE: Systemic lupus erythematosus.

### Mortality of NMOSD

Between 2016 and 2018, the mortality rate was 6•3 deaths per 1000 patients with NMOSD, 106 adults and 4 children died. The average age of death was 56•9 ± 17•4 years. Most of these patients were in the neurological (15•9%) and critical care (28•0%) wards. Deaths are mainly caused by respiratory failure, lung infection, hypoproteinemia, electrolyte disorders, and diabetic ketoacidosis, which account for 78•1% of the total causes of death (Table 4).

**Table 4:** Summary of death.

	2016	2017	2018
**Demographics**			
Age, mean (SD), years	52•9 (15•9)	57•6 (19•8)	60•3 (16•6)
Sex (female), No. (%)	19 (73•1)	27 (71•1)	36 (78•3)
Child/adult, No.	0/26	3/35	1/45
Department*, No. (%)			
Neurology	9 (34•6)	10 (26•3)	18 (39•1)
Critical Care Medicine	7 (26•9)	13 (34•2)	13 (28•3)
Respiratory	1 (3•8)	1 (2•6)	5 (10•9)
Emergency	1 (3•8)	2 (5•3)	2 (4•3)
Infectious Diseases	1 (3•8)	1 (2•6)	2 (4•3)
**Causes**#**, No. (%)**			
Respiratory failure	12 (46•2)	18 (47•4)	21 (45•7)
Lung infection	17 (65•4)	20 (52•6)	29 (63)
Hypoproteinemia	11 (42•3)	13 (34•2)	13 (28•3)
Electrolyte disorders	10 (38•5)	33, 86•8()	33 (71•7)
Diabetic ketoacidosis	5 (19•2)	9 (23•7)	11 (23•9)

⁎ Top 5 departments of discharge.

# The top 5 death related diseases.

## Discussion

Given its complete coverage and emphasis on diagnoses, HQMS serves as a unique and unparalleled resource for determining the incidence of relatively rare diseases such as NMOSD. This is the first nationwide survey performed to evaluate incidence with all age groups of NMOSD in China. We have utilized the regulatory mandated HQMS database which covers all tertiary hospitals in the country. The age and sex adjusted incidence was 0•287 patients per 100,000 individuals, which is higher in adults than that of children (0•347 versus 0•075). This finding is consistent with the reported crude annual incidence of NMOSD in Malaysia (0•39 per 100,000), where 46•9% of prevalent cases are Chinese [22, 23]. The incidence of NMOSD in our study was found to be lower than that reported in Korea (0•73/100,000) [7]. In Western countries, the incidence of NMOSD in Western countries ranges from 0.037 per 100,000 persons in Australia and New Zealand, to 0.132 per 100,000 in Hungary [9, 10, [24], [25], [26], [27]]. In our study, the incidence rate of NMOSD in children was 0•075 per 100,000, which is similar to other countries, including Denmark [28], Catalonia (Spain) [24], and Korea [7]. In a population-based comparative study, the incidence was approximately ten-fold higher (0•73 vs 0•07/100,000) in Martinique (90% Afro-Caribbean) than that in Olmsted County, Minnesota, USA (82% Caucasian) [11]. Likewise, the incidence of NMOSD varies by region and ethnicity. On the basis of these data, our study supports the notion that NMOSD is reported to have ethnic partiality for non-Caucasians, such as Asians.

NMOSD has a high incidence in the 45 to 65-year age range, mainly in females. The female to male ratio is 4•71:1 in our study, this is comparable to the ratio of female to male (6•4:1) reported in Japan [6]. Similarly, the ratio of female to male was observed at 5•75:1 in a single-center hospital-based cohort study in China [29]. Bukhari et al. summarized the ratio of female to male in several NMOSD cohorts of different ethnic groups and found that out of 1864 patients (5•3:1), 1568 of which were female [9].

The highest incidence of NMOSD in China was 0•416/100,000 in Guangxi Province and 0•425/100,000 in Shanxi Province, which are located at latitudes 23° N and 36° N, respectively. Correspondingly, the incidence of NMOSD in Qinghai and Gansu Provinces at equivalent latitudes were 0•358 and 0•244/100,000 (Fig. 2). Consistent with previous studies, a latitude gradient for NMOSD prevalence is not observed [30]. This finding is different from the latitude-related incidence reported for MS, whereas the potential mechanism is believed to be the lack of vitamin D caused by diminished sunlight exposure in high-latitude regions [31]. Our observation further supplements distinct pathologic mechanisms between MS and NMOSD.

The burden of hospitalization in NMOSD patients is gradually decreasing by the year. Given China's health-care reforms, requirements for shortened durations of hospitalization, negotiated price of medications to control disease progression, all partially contribute, to this outcome. Consequently, URBMI and NRCMI covers 68•2% patients with NMOSD. Notably, commercial insurance accounts for a very small percentage of patient coverage. Curiously, hypertension was the most prevalent comorbidity in NMOSD patients. However, the proportion of these patients with hypertension is lower in China than in the United States (14•1% versus 34•4%) [32]. One potential reason for the prevalence of hypertension in the United States is based on the 2017 American College of Cardiology/American Heart Association guideline being twice as high as that based on 2010 Chinese guideline [33]. The high percentage of NMOSD patients with stroke at 7% may be derived from a very high stroke prevalence in China. The National Epidemiological Survey of Stroke in China (NESS-China) shows that the prevalence of stroke in China is 1.1–2.4% [34]. The proportion of anxiety and/or depression in patients with NMOSD is 4.2% in our study. In contrast, the adjusted 1-month prevalence of mood disorders was 6•1%, and anxiety disorders was 5•6% in China [35]. Antibody-mediated diseases, autoimmune conditions were generally high among patients with NMOSD. In this study, autoimmune comorbidities were detected in 10% of patients with NMOSD, which is lower than another recent nationwide population-based study in Sweden (25%) [10]. Consistent with the literature [36], Sjögren's syndrome and SLE are the most common autoimmune comorbidities in NMOSD patients in China. The mortality rate was 6•3 deaths per 1000 patients, with 106 adults and 4 children died during the study period. This aligns with a retrospective study of all patients with NMOSD at two large US-based clinics reporting an annual mortality rate of 6•8 deaths per 1000 patient-years [37].

Several weaknesses of our study are considered and as follows. First, the three-year annual incidence study spans three years which may not be long enough. In addition, MOG-Ab-associated disease has been recognized as a separate disease entity only the past two years. Therefore, there is no identification of MOG-Ab-associated disease in HQMS. Second, we did not have access to outpatient records. As some milder cases of NMOSD may have been missed, the incidence of NMOSD may be underestimated in this study. Third, AQP4-Ab, MOG-Ab test results and MRI findings were not collected in this study. However, the Quality Assurance Physician and coder are required to ensure the accuracy of diagnosis for each hospital record in HQMS. Last, there may be a few patients who passed away outside of the hospitals, so mortality rates may be slightly underestimated.

For the first time, our study captures the incidence for NMOSD in all age groups in almost all Chinese patients. Our study fills the blank of epidemiologic data of the approximately 1•4 billion Chinese population, and enriches global outlook for this disease. The reported disease burden calls for ramping up regional and global efforts to care for NMOSD patients and investing on research for this devastating and pervasive disease.

## Contributors

F.-D.S. and Y.W. conceived and designed this study; F.-D.S., D.-C. T., Z. L., C. Z., M. Y. acquired and analyzed the data; and F.-D.S., D.-C.T., C. Z., drafted the manuscript and prepared the figures; H. G. was involved in statistics analysis; F-D.S. obtained funding; F.-D. S. made critical revisions of the manuscript and important intellectual contributions. All authors reviewed the manuscript.

## Funding/support

This work was supported in part by grants from the National Science Foundation of China (91949208, 91642205, and 81830038); Advanced Innovation Center for Human Brain Protection, Capital Medical University, Beijing.

## Data sharing

The study protocol, statistical analysis plan, and deidentified data that underlie the results of this article will be available for investigators after approval by the Institutional Review Board of China National Clinical Research Center for Neurological Diseases (Beijing, China). Please email the corresponding author for more information.

## Declaration of Competing Interest

None.

## Appendix Supplementary materials

Image, application 1

Image, application 2

Image, application 3
