# Corrigendum to ‘Incidence of neuromyelitis optica spectrum disorder (NMOSD) in China: A national population-based study’

**DOI:** 10.1016/j.lanwpc.2021.100118

**Published:** 2021-03-02

**Authors:** De-Cai Tian, Zixiao Li, Meng Yuan, Chengyi Zhang, Hongqiu Gu, Yongjun Wang, Fu-Dong Shi

**Affiliations:** a China National Clinical Research Center for Neurological Diseases, Beijing Tiantan Hospital, Capital Medical University, Beijing 100070, China; b Department of Neurology, Tianjin Neurological Institute, Tianjin Medical University General Hospital, Tianjin 300052, China

The authors regret an error in citation in the following sentence in section “Introduction”: “and 1•94/100,000 in Malaysia.^8^”

The correct reference should be: [1]

The authors would like to apologise for any inconvenience caused.
