# Supplementary material for: Incidence of neuromyelitis optica spectrum disorder (NMOSD) in China: A national population-based study
Source: Lancet Reg Health West Pac. 2020 Sep 6;2:100021. doi: 10.1016/j.lanwpc.2020.100021 (PMC8315565; doi:10.1016/j.lanwpc.2020.100021)

**Web Appendix Material**

Incidence of neuromyelitis optica spectrum disorder (NMOSD) in China: a national population-based study

Section 1 Data Collection and Quality Control .................................................................................................2

Section 2 Analysis protocol ...............................................................................................................................16

Appendix Table 1 The number of tertiary hospitals involved in each province in this study............... ............17

Appendix Figure 1 The geographical distribution of 1665 tertiary hospitals in this study ................................18

**Section 1** **Data Collection and Quality Control of HQMS**

**1. Overview of The Hospital Quality Monitoring System (HQMS) in China**

The Hospital Quality Monitoring System (HQMS) is an online data collection system launched for medical care measurement and improvement by the Chinese government in 2011. The system is designed to link hospital information systems and to upload a common dataset of information from all inpatient medical records. The HQMS covers all tertiary hospitals over the country, and inpatient data from each tertiary hospital can automatically electronically captured and uploaded. The basic information of HQMS is comparable to the claimant data required by the Centers for Medicare & Medicaid Services in the US[^1^](#_ENREF_1).

The HQMS contains some basic information about patient hospitalization, but lacks clinical information about treatments, patient indications, and contraindications, including: patients' social ID, demographic characteristics, length of stay in hospital, principal admission diagnosis, discharge diagnosis (principal ones and comorbidities), special treatments (procedures and surgeries), patient outcomes (death, non-recovery, improvement, or complete recovery), in-hospital infection (whether or not), transfusion, and the fee for the inpatient management[^2-4^](#_ENREF_2). The HQMS’s nationwide and unique patient identification system allows for information linkage across barriers of time and locale; a relevant and necessary feature given China’s enormous population mobility.

**2. Data Collection Standard for Medical Record Homepage in Each Tertiary Public Hospitals (related to this study)**

| **No.** | **Data Collection item** | **Code** | **Type** | **Length** | **Remark** |
| --- | --- | --- | --- | --- | --- |
|  | Institution code | A01 | Character | 22 | Refers to the institution code on the practice license of medical institution |
|  | Medical institution name | A02 | Character | 80 | Refers to the name of the medical institution where the patient is hospitalized, according to the name of the institution registered on the ‘The Practice License of Medical Institution’ |
|  | Medical record number | A48 | Character | 50 |  |
|  | Number of hospitalizations | A49 | Number | 4 | An integer greater than 0 |
|  | Admission time | B12 | Date |  | Format yyyy-MM-dd HH: mm: ss; the admission time should not be later than the discharge time |
|  | Discharge time | B15 | Date |  | Format yyyy-MM-dd HH: mm: ss |
|  | Health card number | A47 | Character | 50 | Fill in the health card number in the area where the "Health Card of China" has been issued uniformly, or fill in "-" in the areas where the "Health Card" has not been issued. |
|  | Medical payment method | A46C | Character | 3 | Threshold reference range RC032. |
|  | Name | A11 | Character | 40 |  |
|  | Gender | A12C | Number | 1 | Threshold reference range RC001 |
|  | Date of birth | A13 | Date | 10 | Format yyyy-MM-dd |
|  | Age (year old) | A14 | Number | 3 | The age at which a patient is admitted refers to the calendar age calculated on the calendar when the patient is admitted to the hospital. An integer greater than or equal to 0 |
|  | Nationality | A15C | Character | 40 |  |
|  | Marital status | A21C | Character | 1 | Threshold reference range RC002 |
|  | Occupation | A38C | Character | 2 | Threshold reference range RC003 |
|  | Ethnic group | A19C | Character | 2 | Threshold reference range RC035 |
|  | Identity number | A20 | Character | 18 | Inpatients must fill in 15 or 18 ID numbers when they are admitted |
|  | Birthplace | A22 | Character | 200 |  |
|  | Native province (autonomous region, municipality) | A23C | Character | 50 | Threshold reference range RC036 |
|  | Registered residence address | A24 | Character | 200 |  |
|  | Registered permanent residence zip code | A25C | Character | 6 | 6 digits |
|  | Current address | A26 | Character | 200 |  |
|  | Current address phone number | A27 | Character | 40 |  |
|  | Current address zip code | A28C | Character | 6 | 6 digits |
|  | Work unit and address | A29 | Character | 200 |  |
|  | Work unit phone number | A30 | Character | 20 |  |
|  | Work unit zip code | A31C | Character | 6 | 6 digits |
|  | Contact name | A32 | Character | 40 |  |
|  | Contact relationship | A33C | Character | 1 | Threshold reference range RC033 |
|  | Contact address | A34 | Character | 200 |  |
|  | Admission way | B11C | Character | 1 | Threshold reference range RC026 |
|  | Admission department | B13C | Character | 6 | Threshold reference range RC023 |
|  | Admission ward | B14 | Character | 30 |  |
|  | Transfer department | B21C | Set | Multiple choices | [Threshold reference range RC023；When passing through multiple departments, the values are separated by commas.](#RC023) |
|  | Discharge department | B16C | Character | 6 | Threshold reference range RC023 |
|  | Discharge ward | B17 | Character | 30 |  |
|  | Length of stay in hospital | B20 | Number | 6 | An integer greater than 0. The admission time and discharge time are only calculated for one day. For example: admitted on June 12, 2018, and discharged on June 15, 2018, the length of stay in hospital is 3 days. |
|  | Outpatient (emergency) diagnosis code | C01C | Character | 20 | Use the International Classification of Diseases, 10th revision (ICD-10) |
|  | Outpatient (emergency) diagnosis | C02N | Character | 100 | Use ICD-10 and the corresponding diagnosis name |
|  | Primary discharge diagnosis code | C03C | Character | 20 | Use ICD-10 |
|  | Primary discharge diagnosis | C04N | Character | 100 | Use ICD-10 and the corresponding diagnosis name |
|  | Admission condition of primary discharge diagnosis | C05C | Character | 1 | Threshold reference range RC027 |
|  | Other discharge diagnosis code | C06x01C | Character | 20 | Use ICD-10 |
|  | Other discharge diagnosis | C07x01N | Character | 100 | Use ICD-10 and the corresponding diagnosis name |
|  | Admission condition of other discharge diagnosis | C08x01C | Character | 1 | Threshold reference range RC027 |
|  | Pathological diagnosis code | C09C | Character | 20 | The Second Edition of the International Classification of Diseases for Oncology (M code) is used; it is required when the first letter of the primary diagnosis ICD code is C or D00-D48. |
|  | Pathological diagnosis name | C10N | Character | 100 | Second Edition of the International Classification of Diseases for Oncology (M code) and the pathological name corresponding to the code; the primary diagnosis ICD code must be C or D00-D48. |
|  | Pathology number | C11 | Character | 50 | Required if there is a pathological diagnosis code. |
|  | Drug allergy or not | C24C | Character | 1 | Threshold reference range RC037 |
|  | Allergic drug name | C25 | Character | 200 | Required if " Drug Allergy" is "Yes"; multiple drugs are separated by commas |
|  | Chief director | B22 | Character | 40 |  |
|  | Chief (co-chief) doctor | B23 | Character | 40 |  |
|  | Doctor-in-charge | B24 | Character | 40 |  |
|  | Resident doctor | B25 | Character | 40 |  |
|  | Nurse-in- charge | B26 | Character | 40 |  |
|  | Intern doctor | B28 | Character | 40 |  |
|  | Coder | B29 | Character | 40 |  |
|  | Medical record quality | B30C | Character | 1 | Threshold reference range RC011 |
|  | Quality Control Doctor | B31 | Character | 40 |  |
|  | Quality Control Nurse | B32 | Character | 40 |  |
|  | Quality control date | B33 | Date |  | Format yyyy-MM-dd |
|  | Autopsy of dead patients | C34C | Character | 1 | Threshold reference range RC016 |
|  | ABO blood group | C26C | Character | 1 | Threshold reference range RC030 |
|  | Rh blood group | C27C | Character | 1 | Threshold reference range RC031 |
|  | Age under 1 year (day) | A16 | Number | 3 | Fill in the number of days of actual age. Fill in when the age is less than 1 year old. The age value A14 should be 0. The value range is greater than or equal to 0 and less than 365. The time of admission minus the date of birth is rounded up to 0. |
|  | Newborn birth weight (g) | A18x01 | Number | 6 | The weight of a newborn should be accurate to 10 grams; the weight should be weighed within one hour after the live birth. 1. Fill in the medical records of the mother and the newborn. From birth to 28 days is the neonatal period. For the weight of the twins and above, continue to fill in the following birth weight. 2. Weight range of newborn: 100g-9999g. When the maternal primary diagnosis or other diagnostic codes contain Z37.0, Z37.2, Z37.3, Z37.5, Z37.6 codes, the birth weight of the newborn must be entered. |
|  | Rehospitalization plan within 31 days after discharge or not | B36C | Number | 1 | Threshold reference range RC028. Refers to the patient's rehospitalization arrangements within 31 days after the hospitalization and discharge. If there is a rehospitalization plan, you need to fill in the purpose, for example: performing a second operation. |
|  | Purpose of 31-Day Rehospitalization | B37 | Character | 100 | Required if rehospitalization plan within 31 days after discharge is Yes. |
|  | Discharge way | B34C | Character | 1 | Threshold reference range RC019；Refer to the way the patient was hospitalized and discharged, fill in the corresponding Arabic numbers. |
|  | Total hospitalization fee | D01 | Number | (11,2) | The total hospitalization fee is required and greater than 0; the total fee is greater than or equal to the sum of the sub-items |
|  | Self-paying fee in the total hospitalization fee | D09 | Number | (10,2) | Less than or equal to total hospitalization fee |
|  | 1.Service fee of general medical treatment | D11 | Number | (10,2) |  |
|  | 2.Operating fee of general medical treatment | D12 | Number | (10,2) |  |
|  | 3.Nursing fee | D13 | Number | (10,2) |  |
|  | 4.Integrated Medical Services fee | D14 | Number | (10,2) |  |
|  | 5.Pathology diagnosis fee | D15 | Number | (10,2) |  |
|  | 6.Laboratory diagnosis fee | D16 | Number | (10,2) |  |
|  | 7.Fee of imaging diagnosis | D17 | Number | (10,2) |  |
|  | 8Clinical diagnosis fee | D18 | Number | (10,2) |  |
|  | 9Non-surgical treatment items fee | D19 | Number | (10,2) |  |
|  | 10.Surgery treatment fee | D20 | Number | (10,2) |  |
|  | 11Rehabilitation fee | D21 | Number | (10,2) |  |
|  | 12.Chinese medicine fee | D22 | Number | (10,2) |  |
|  | 13Western medicine fee | D23 | Number | (10,2) |  |
|  | 14.Chinese patent medicine fee | D24 | Number | (10,2) |  |
|  | 15Chinese herbal medicine fee | D25 | Number | (10,2) |  |
|  | 16.Blood fee | D26 | Number | (10,2) |  |
|  | 17.Albumin products fee | D27 | Number | (10,2) |  |
|  | 18.Globulin products fee | D28 | Number | (10,2) |  |
|  | 19. Blood clotting factors fee | D29 | Number | (10,2) |  |
|  | 20.Cytokines products fee | D30 | Number | (10,2) |  |
|  | 21Disposable medical materials for examination fee | D31 | Number | (10,2) |  |
|  | 22Disposable medical materials for treatment fee | D32 | Number | (10,2) |  |
|  | 23.Disposable medical materials for surgery fee | D33 | Number | (10,2) |  |
|  | 24.Other fees | D34 | Number | (10,2) |  |

# 2.2 *Data element value field code*

### 2.2.1 *RC001 Gender code*

| **Value** | **Name** | **Remark** |
| --- | --- | --- |
| 0 | 0-Unknown sex |  |
| 1 | 1-Male |  |
| 2 | 2-Female |  |
| 9 | 9-Unspecified gender |  |
| This code comes from the national standard < Classification and codes of basic personal information> (GB/T 2261.1-2003) | | |

### 2.2.2 RC002 Marital status code

| **Value** | **Name** | **Remark** |
| --- | --- | --- |
| 1 | 1-Unmarried |  |
| 2 | 2-Married |  |
| 3 | 3-Widowed |  |
| 4 | 4-Divorced |  |
| 9 | 9-Other |  |
| This code comes from the national standard <Classification and codes of basic personal information> (GB/T 2261.1-2003) | | |

### 2.2.3 *RC003 Occupation code*

| **Value** | **Name** | **Remark** |
| --- | --- | --- |
| 11 | 11-National civil Servant |  |
| 13 | 13-Professional and technical personnel |  |
| 17 | 17-Staff |  |
| 21 | 21-Corporate executives |  |
| 24 | 24-Workers |  |
| 27 | 27-Farmers |  |
| 31 | 31-Student |  |
| 37 | 37-Serving soldier |  |
| 51 | 51-Freelance |  |
| 54 | 54-Self-employed |  |
| 70 | 70-Unemployed |  |
| 80 | 80-Retired |  |
| 90 | 90-Other |  |
| This code comes from the national standard<Classification and codes of basic personal information>(GB/T2261.4) | | |

### 2.2.4 *RC011 Medical record quality code*

| **Value** | Name | Remark |
| --- | --- | --- |
| 1 | 1-A | Good |
| 2 | 2-B | General |
| 3 | 3-C | Bad |

### 2.2.5 *RC019 Discharge way code*

| **Value** | **Name** |
| --- | --- |
| 1 | Leave the hospital with a doctor's advice |
| 2 | Transfer with a doctor's advice |
| 3 | Transfer to community health service institutions/township health centers with a doctor's advice |
| 4 | Leave the hospital without a doctor's advice |
| 5 | Dead |
| 9 | Other |

### 2.2.6 *RC023 Department code (<List of diagnosis and treatment subjects in medical institutions>)*

| **Value** | **Name** |
| --- | --- |
| 01 | Preventive Medicine Department |
| 02 | General Medicine Department |
| 03 | Internal Medicine Department |
| 0301 | Respiratory Department |
| 0302 | Gastroenterology Department |
| 0303 | Neurology Department |
| 0304 | Cardiology Department |
| 0305 | Hematology Department |
| 0306 | Nephrology Department |
| 0307 | Endocrinology Department |
| 0308 | Immunology Department |
| 0309 | Allergy Department |
| 0310 | Geriatric Department |
| 0311 | Other |
| 04 | Surgery |
| 0401 | General Surgery |
| 040101 | Liver Transplantation |
| 040102 | Pancreatic Transplantation |
| 040103 | Small Bowel Transplantation |
| 0402 | Neurological Surgery |
| 0403 | Orthopedics Department |
| 0404 | Urology Surgery |
| 040401 | Kidney Transplantation |
| 0405 | Thoracic Surgery |
| 040501 | Lung Transplantation |
| 0406 | Cardiac and Macrovascular Surgery |
| 040601 | Heart Transplantation |
| 0407 | Department of Burn |
| 0408 | Plastic Surgery |
| 0409 | Other |
| 05 | Obstetric and Gynecologic Department |
| 0501 | Gynecology Department |
| 0502 | Obstetrics Department |
| 0503 | Family Planning Department |
| 0504 | Eugenics Discipline Department |
| 0505 | Reproductive Health and Infertility Department |
| 0506 | Other |
| 06 | Women's Health Care Department |
| 0601 | Adolescent Health Care Department |
| 0602 | Perinatal Health Care Department |
| 0603 | Climacteric Health Care Department |
| 0604 | Women's Mental Health Department |
| 0605 | Women's Nutrition Department |
| 0606 | Other |
| 07 | Pediatrics Department |
| 0701 | Neonatal Pediatrics Department |
| 0702 | Pediatric Infectious Diseases Department |
| 0703 | Pediatric Gastroenterology Department |
| 0704 | Pediatric Respiratory Department |
| 0705 | Pediatric Cardiology Department |
| 0706 | Pediatric Nephrology Department |
| 0707 | Pediatric Hematology Department |
| 0708 | Pediatric Neurology Department |
| 0709 | Pediatric Endocrinology Department |
| 0710 | Pediatric Genetic Diseases Department |
| 0711 | Pediatric Immunology Department |
| 0712 | Other |
| 08 | Pediatric Surgery |
| 0801 | Pediatric General Surgery |
| 0802 | Pediatric Orthopedics |
| 0803 | Pediatric Urology |
| 0804 | Pediatric Cardiothoracic Surgery |
| 0805 | Pediatric Neurological Surgery |
| 0806 | Other |
| 09 | Child Healthcare Department |
| 0901 | Child Growth & Development Department |
| 0902 | Child Nutrition Department |
| 0903 | Child Mental Health Department |
| 0904 | Facial Features and Health Care for Children Department |
| 0905 | Child Rehabilitation Department |
| 0906 | Other |
| 10 | Ophthalmology Department |
| 11 | Otolaryngology Department |
| 1101 | Otology Department |
| 1102 | Rhinology Department |
| 1103 | Pharyngology Department |
| 1104 | Other |
| 12 | Stomatology Department |
| 1201 | Stomatology Department |
| 1202 | Oral and Maxillofacial Surgery Department |
| 1203 | Orthodontic Department |
| 1204 | Prosthodontics Department |
| 1205 | Oral Preventive Health Department |
| 1206 | Other |
| 13 | Dermatology Department |
| 1301 | Dermatology Department |
| 1302 | Sexually Transmitted Diseases Department |
| 1303 | Other |
| 14 | Medical Cosmetology Department |
| 15 | Psychiatry Department |
| 1501 | Psychiatric Department |
| 1502 | Mental Health Department |
| 1503 | Drug Dependence Department |
| 1504 | Psychiatric Rehabilitation Department |
| 1505 | Community Prevention and Control Section Department |
| 1506 | Clinical Psychology Department |
| 1507 | Judicial Psychiatric Department |
| 1508 | Other |
| 16 | Infectious Diseases Department |
| 1601 | Intestinal Infectious Diseases Department |
| 1602 | Respiratory Infectious Diseases Department |
| 1603 | Hepatitis Department |
| 1604 | Insect-borne Infectious Diseases Department |
| 1605 | Animal Infectious Diseases Department |
| 1606 | Worm Diseases Department |
| 1607 | Other |
| 17 | Tuberculosis Department |
| 18 | Endemic Department |
| 19 | Oncology Department |
| 20 | Emergency Department |
| 21 | Rehabilitation Medicine Department |
| 22 | Sports Medicine Department |
| 23 | Occupational Disease Department |
| 2301 | Occupational Toxicology Department |
| 2302 | Pneumoconiosis Department |
| 2303 | Radiology Department |
| 2304 | Physical Factor Damage Department |
| 2305 | Occupational Health Care Unit |
| 2306 | Other |
| 24 | Hospice care Department |
| 25 | Special Medicine and Military Medicine Department |
| 26 | Anesthesiology Department |
| 27 | Pain Department |
| 28 | Critical Medicine Department |
| 30 | Medical Laboratory |
| 3001 | Clinical Humoral and Hematological Department |
| 3002 | Clinical Microbiology Department |
| 3003 | Clinical Biochemical Laboratory Department |
| 3004 | Clinical Immunology and Serology Department |
| 3005 | Clinical Cell and Molecular Genetics Department |
| 3006 | Other |
| 31 | Pathology Department |
| 32 | Medical Mm aging Department |
| 3201 | X-ray Diagnosis Department |
| 3202 | CT Diagnostic Department |
| 3203 | Magnetic Resonance Imaging Diagnosis Department |
| 3204 | Nuclear Medicine Department |
| 3205 | Ultrasonic Diagnosis Department |
| 3206 | ECG Laboratory |
| 3207 | EEG Laboratory |
| 3208 | Neuromuscular Electrography Laboratory |
| 3209 | Invasive Department and Medical Imagery |
| 3210 | Radiotherapy Department |
| 3211 | Other |
| 50 | Traditional Chinese Medicine Department |
| 5001 | Internal Medicine Department |
| 5002 | Surgery Department |
| 5003 | Maternity Department |
| 5004 | Pediatrics Department |
| 5005 | Dermatology Department |
| 5006 | Ophthalmology Department |
| 5007 | Otolaryngology Department |
| 5008 | Stomatology Department |
| 5009 | Oncology Department |
| 5010 | Orthopedics Department |
| 5011 | Proctology Department |
| 5012 | Geriatric Department |
| 5013 | Acupuncture and moxibustion Department |
| 5014 | Massage Department |
| 5015 | Rehabilitation Medicine Department |
| 5016 | Emergency Department |
| 5017 | Preventive Medicine Department |
| 5018 | Other |
| 51 | Ethnic Medicine Department |
| 5101 | Uygur Medicine Department |
| 5102 | Tibetan Medicine Department |
| 5103 | Mongolian Medicine Department |
| 5104 | Yi Medicine Department |
| 5105 | Dai Medicine Department |
| 5106 | Other |
| 52 | Integrated TCM & Western Medicine Department |
| 69 | Other Business Department |

### 2.2.10 *RC026 Admission way code*

| **Value** | **Name** |
| --- | --- |
| 1 | 1-Emergency |
| 2 | 2-Outpatient |
| 3 | 3-Transfered from other medical institutions |
| 9 | 9-Other |

### 2.2.7 *RC027 Admission condition code*

| **Value** | **Name** |
| --- | --- |
| 1 | 1-Yes |
| 2 | 2-Clinically undetermined |
| 3 | 3-Unknown |
| 4 | 4-No |

### 2.2.8 *RC028 Rehospitalization plan within 31 days after discharge code*

| **Value** | **Name** |
| --- | --- |
| 1 | No |
| 2 | Yes |

### 2.2.9 *RC030 ABO blood group code*

| **Value** | **Name** |
| --- | --- |
| 1 | Type A |
| 2 | Type B |
| 3 | Type O |
| 4 | Type AB |
| 5 | Unknown |
| 6 | Not checked |

### 2.2.10 *RC031 Rh blood group code*

| **Value** | **Name** |
| --- | --- |
| 1 | Negative |
| 2 | Positive |
| 3 | Unknown |
| 4 | Not checked |

### 2.2.11 *RC032 Medical payment methods code*

| **Value** | **Name** |
| --- | --- |
| 1.1 | 1.1- UEBMI: The Urban Employee Basic Medical Insurance (local) |
| 1.2 | 1.2- UEBMI: The Urban Employee Basic Medical Insurance (externa) |
| 2.1 | 2.1- URBMI: The Rural Resident Basic Medical Insurance (local) |
| 2.2 | 2.2- URBMI: The Rural Resident Basic Medical Insurance (externa) |
| 3.1 | 3.1-NRCMI: The New Rural Cooperative Medical Insurance (local) |
| 3.2 | 3.2-NRCMI: The New Rural Cooperative Medical Insurance (externa) |
| 4 | 4-Poverty Relief |
| 5 | 5-Commercial Health Insurance |
| 6 | 6-All public health service |
| 7 | 7-Fully self-funded |
| 8 | 8-Other Social Insurance |
| 9 | 9-Other |

### 2.2.12 *RC033 Contact relationship code*

| **Value** | **Name** |
| --- | --- |
| 0 | 0-Self or the head of household |
| 1 | 1-Spouse |
| 2 | 2-Son |
| 3 | 3-Daughter |
| 4 | 4-Grandchildren |
| 5 | 5-Parents |
| 6 | 6-Grandparents |
| 7 | 7-Brothers and sisters |
| 8 | 8-Colleagues and classmates |
| 9 | 9-Other |
| This code refers to the national standard of "Family relationship Code" (GB/T4761) | |

### 2.2.13 *RC035 Ethnic Group code*

| **Value** | **Name** |
| --- | --- |
| 1 | 1-Han |
| 2 | 2-Mongolian |
| 3 | 3-Hui |
| 4 | 4-Tibetan |
| 5 | 5-Uygur |
| 6 | 6-Miao |
| 7 | 7-Yi |
| 8 | 8-Zhuang |
| 9 | 9-Buyei |
| 10 | 10-Korean |
| 11 | 11-Manchu |
| 12 | 12-Dong |
| 13 | 13-Yao |
| 14 | 14-Bai |
| 15 | 15-Tujia |
| 16 | 16-Hani |
| 17 | 17-Kazakh |
| 18 | 18-Dai |
| 19 | 19-Li |
| 20 | 20-Lisu |
| 21 | 21-Va |
| 22 | 22-She |
| 23 | 23-Gaoshan |
| 24 | 24-Lahu |
| 25 | 25-Shui |
| 26 | 26-Dongxiang |
| 27 | 27-Naxi |
| 28 | 28-Jingpo |
| 29 | 29-Kirgiz |
| 30 | 30-Tu |
| 31 | 31-Daur |
| 32 | 32-Mulao |
| 33 | 33-Qiang |
| 34 | 34-Bulang |
| 35 | 35-Salar |
| 36 | 36-Maonan |
| 37 | 37-Gelao |
| 38 | 38-Xibe |
| 39 | 39-Achang |
| 40 | 40-Pumi |
| 41 | 41-Tajik |
| 42 | 42-Nu |
| 43 | 43-Uzbek |
| 44 | 44-Russ |
| 45 | 45-Ewenki |
| 46 | 46-De’ang |
| 47 | 47-Baoan |
| 48 | 48-Yugu |
| 49 | 49-Jing |
| 50 | 50-Tatar |
| 51 | 51-Dulong |
| 52 | 52-Oroqen |
| 53 | 53-Hezhe |
| 54 | 54-Mamba |
| 55 | 55-Lhoba |
| 56 | 56-Jinuo |
| 66 | 66-Other |
| 99 | 99-Foreign nationals |

### 2.2.14 *RC036 Administrative division*

| **Value** | **Name** |
| --- | --- |
| 1 | 1-Beijing |
| 2 | 2-Tianjin |
| 3 | 3-Hebei |
| 4 | 4-Shanxi |
| 5 | 5-Inner Mongolia |
| 6 | 6-Liaoning |
| 7 | 7-Jilin |
| 8 | 8-Heilongjiang |
| 9 | 9-Shanghai |
| 10 | 10-Jiangsu |
| 11 | 11-Zhejiang |
| 12 | 12-Anhui |
| 13 | 13-Fujian |
| 14 | 14-Jiangxi |
| 15 | 15-Shandong |
| 16 | 16-Henan |
| 17 | 17-Hubei |
| 18 | 18-Hunan |
| 19 | 19-Guangdong |
| 20 | 20-Guangxi |
| 21 | 21-Hainan |
| 22 | 22-Sichuan |
| 23 | 23-Chongqing |
| 24 | 24-Guizhou |
| 25 | 25-Yunnan |
| 26 | 26-Tibet |
| 27 | 27-Shaanxi |
| 28 | 28-Gansu |
| 29 | 29-Qinghai |
| 30 | 30-Ningxia |
| 31 | 31-Xinjiang |
| 32 | 32-Taiwan |
| 33 | 33-Hong Kong |
| 34 | 34-Macau |

### 2.3.15 *RC037 Drug allergy*

| **Value** | **Name** |
| --- | --- |
| 1 | 1-Yes |
| 2 | 2-No |

## 3. Quality Control

## 3.1 Basic rules of data quality control

1. Data type should be compatible with interface standards;
2. The data length does not exceed the maximum length specified by the interface standard;
3. The data accuracy should be compatible with the interface standard. For example, if the length tag is "(10,2)", the length is 10 digits, and 2 decimal places are reserved;
4. If the interface standard specifies threshold reference range, the data must be within the range;
5. If the interface standard specifies a unit, the data must match the unit;
6. If the data type is numeric, the data must be greater than or equal to 0 if it is not blank;
7. The field names of the data collection items are consistent with the requirements of the interface standard;

h) None of the "required" data can be blank or NULL. If there is no data or data is not applicable, fill in English dash "-".

i) “Required depending on the conditions” is not a required term. When the condition is met, the data of the "required depending on the conditions" cannot be blank or NULL.

## 3.2 Data Association Verification Rules

| **Verification Rules** | **Verification instructions** | **Related parameter description** |
| --- | --- | --- |
| Verify age and date of birth | Age equals admission time minus birthdate (the margin of error is 1 year) | Admission time (B12)  Date of birth (A13)  Age (A14) |
| Verify the relationship between admission time, discharge time and surgical operation time | Admission time ≤ discharge time;  (Admission time-1 day) ≤ surgical operation time ≤ discharge time; | Admission time (B12)  Discharge time (B15)  primary surgical operation time (C16x01)  other surgical operation time 1-40 (C37x01-C37x40) |
| Verify diagnosis code range | Outpatient (emergency) diagnosis code, primary and other discharge diagnosis code should be in the range of codes beginning with A to U and Z; excluding codes beginning with letters V, W, X, and Y | Outpatient (emergency) diagnosis code (C01C)  primary discharge diagnosis code(C03C)  other discharge diagnosis code 1-40 (C06x01C-C06x40C) |
| Verify the diagnosis code for neonatal birth injury and neonatal age (change on May 8, 2019) | When P10~P15 appears in the outpatient (emergency) diagnosis code, primary or other discharge diagnosis code, the admission time minus the date of birth must be less than 365 days, and the age (days) under 1 year old must be less than 365 days | Outpatient (emergency) diagnosis code(C01C)  primary discharge diagnosis code (C03C)  Other discharge diagnosis code 1-40 (C06x01C-C06x40C)  Admission time (B12)  Birthdate (A13)  Age (days) under 1 year old (A16) |
| Verify the integrity of the outcome coded by delivery and delivery (abortion) | When O80-O84 appears in the primary or other discharge diagnosis code, and no O00-O08 appears in the abortive outcome code, the other discharge diagnosis code must have the delivery outcome code Z37 | primary discharge diagnosis code (C03C)  Other discharge diagnosis code 1-40 (C06x01C-C06x40C) |
| Verify the integrity of newborn birth weight | When the primary or other discharge diagnosis code are Z37.0, Z37.2, Z37.3, Z37.5, Z37.6, or the age (days) under 1 year old is less than 28 days, or the admission time minus the birth date is less than or equal to 28 days, the birth weight of the newborn should not be blank. | Newborn birth weight (A18x01)  Primary discharge diagnosis code (C03C)  Other discharge diagnosis code 1-40 (C06x01C-C06x40C)  Age (days) under 1 year old (A16)  Admission time (B12)  Date of birth (A13) |
| Verify the integrity of newborn admission weight | When the age (days) under 1 year old ≤ 28 days, or admission time minus date of birth≤ 28 days, the neonatal admission weight should not be blank. | Newborn admission weight (A17)  Age (days) under 1 year old (A16)  Admission time (B12)  Date of birth (A13) |
| Verify the integrity of age (days) under 1 year old | When the age is under 1 year old, that is, the age is 0, the age (days) under 1 year old should not be blank. | Age (days) under 1 year old (A16)  Age (A14) |
| Verify the relationship between the total fee of hospitalization and the sum of sub-items | The total fee of hospitalization is required and greater than 0; the total fee of hospitalization is greater than or equal to the sum of the sub-items  D01≥(D11+D12+D13+D14+D15+D16+D17+D18+D19+D20+D21+D22+D23+D24+D25+D26+D27+D28+D29+D30+D31+D32+D33+D34)； | Total fee of hospitalization (D11)  General treatment operation fee (D12)  Nursing fee (D13)  Other fee for Comprehensive Medical Services (D14)  Pathological diagnosis fee (D15)  Laboratory diagnosis fee (D16)  Imaging diagnosis fee (D17)  Clinical diagnosis item fee (D18)  Non-surgical treatment item fee (D19)  Surgical treatment fee (D20)  Rehabilitation fee (D21)  Chinese medicine treatment fee (D22)  Western medicine fee (D23)  Chinese patent medicine fee (D24)  Chinese herbal medicine fee (D25)  Blood fee (D26)  Albumin products fee(D27)  Globulin products fee(D28)  Clotting factor products fee(D29)  Cytokine products fee(D30)  Disposable inspection medical supply fee(D31)  Disposable treatment medical supply fee(D32)  Disposable surgery medical supply fee(D33)  Other fees (D34) |
| Verify the relationship between total hospital fee and self-paying fee from total hospitalization fee | The total hospitalization fee must be greater than or equal to self-paying fee out of total hospitalization fee D01≥D09 | Total hospitalization fee(D01)  self-paying fee out of total hospitalization fee (D09) |
| Verify the relationship between nonsurgical treatment item fee, clinical physiotherapy fee | The nonsurgical treatment item fee must be greater than or equal to the clinical physiotherapy fee D19≥D19x01 | Nonsurgical treatment item fee (D19)  Clinical physiotherapy fee (D19x01) |
| Verify the relationship between surgical treatment fee, anesthesia fee and surgery fee | The surgical treatment fee must be greater than or equal to the sum of anesthesia fee and surgery fee D20≥(D20x01+D20x02) | Surgical treatment fee (D20)  Anesthesia fee (D20x01)  Surgery fee (D20x02) |
| Verify the relationship between western medicine fee and antibacterial drug fee | The western medicine fee must be greater than or equal to the antibacterial drug fee  D23≥D23x01 | Western medicine fee (D23)  Antibacterial drug fee (D23x01) |
| Verify the quality control (QC) date range | The QC date cannot be earlier than the admission time | QC date (B33)  Admission time (B12) |
| Verify the history of drug allergies and names of allergic drugs | If there is a drug allergy, the name of the allergic drug is required. | drug allergy or not(C24C) 2=yes  Name of the allergic drug (C25) |
| Verify the relationship between discharge way and name of the doctor's order to transfer to other hospitals or the community health service/ township hospitals | The discharge way is required for patients transferred by doctor's order to other hospitals or community health service / township hospitals. | Discharge way (B34C)  2= (transferred by doctor's order to other hospitals), 3= (transferred by doctor's order to community health service / township hospitals)  Name of the doctor's order to transfer to other hospitals or the community health service/ township hospitals (B35) |
| Verify the relationship between whether there is a readmission plan within 31 days after discharge or not and the purpose of the 31-day hospital readmission plan | If there is a readmission plan within 31 days after discharge, the purpose of 31-day hospital readmission is required. | readmission plan within 31 days after discharge or not (B36C) 2=yes  Purpose of 31-day hospital readmission (B37) |
| Verify the relationship between the primary discharge diagnosis code and the pathological diagnosis code, pathological diagnosis name and pathology number at discharge | Required for cancer patients. When the initial letter of the primary discharge diagnosis code is C or D00-D10 or D45-D47, the pathological diagnosis code, pathological diagnosis name and pathology number should not be empty. | Primary discharge diagnosis code (C03C)  Pathological diagnosis code (C09C)  Pathological diagnosis name (C10N)  Pathology number (C11) |
| Verify the relationship between primary discharge diagnosis code and both the code and name of external causes of injury and poisoning | Required for patients with poisoning; when the initial letter of the primary discharge diagnosis code is S or T, the code and name of external causes of the injury and poisoning should not be empty. | The primary discharge diagnosis code (C03C)  Code of external causes of the injury and poisoning (C12C)  Name of external causes of the injury and poisoning name (C13N) |
| Verify the ID number | The ID card number needs to comply with the provisions of the National Standard GB 11643-1999 of the People's Republic of China on citizenship number. | ID card number (A20) |

**Section 2 Analysis protocol**

**1. Basic Requirements**

Accord to "field definition" in section 2 HQMS interface standard and "value threshold range" in its notes.

1.1 *Search time range*

According to the time of discharge (B5): 2016.1.1-2018.12.31.

1.2 *Analysis related keywords*

Neuromyelitis optical spectrum disorder (NMOSD).

1.3 *New variable generated from the previous criterion*

| **New variable** | **Formula** | **Meaning and threshold** |
| --- | --- | --- |
| C03C | C03C | **Primary diagnosis code:** National Classification 2.0 Disease Diagnosis Code (ICD-10) |
| C06 | The collection of these 40 variables from C06x01C toC06x40C | **All other diagnoses：**the value threshold is the disease ICD-10 code (composed of a letter and a string of numbers) |

1.4 *Specification of results required*

Mean ± standard deviation, 95% CI, median± IQR, P50 (P25, P75).

**2. Identify the Study Population**

2.1 *Search criteria*: primary diagnosis code (C03C) or other discharge diagnosis code (C06x01C-C06x40C).

2.2 *Newly generated variables*

Diagnosis code refers to international Classification of Diseases, Tenth revision (ICD-10) code (China National Edition 2.0).

| **New variable** | **ICD-10 code** | **Meaning** |
| --- | --- | --- |
| uIDD | G37.5 or G37.8 or G37.9 or G04.800 | Unclassed idiopathic central nervous system demyelinating disease |
| ADEM | G36.801 or G04.001 | Acute disseminated encephalomyelitis |
| ATM | G36.901 or G37.300 or G04.902 or G04.909 or G04.910 | Acute Myelitis |
| ON | H46 | Optic neuritis |
| MS | G35.0 | Multiple sclerosis |
| NMOSD | G36.000 | Neuromyelitis optica [Devic] |
|  | G36.000x002 | Neuromyelitis optica Spectrum disorder |
| CIS | G37.805 | Clinical isolated syndrome |

2.3 *The same person definition*: Generate new variables based on name, age, and ID.

**3.Steps of Analysis in NMOSD**

3.1 *Data source description*

Number of hospitals reported by provinces showing the number of hospitals related to the total study population

| Province | Number of HQMS hospitals reported | Number of consecutively reported hospitals |
| --- | --- | --- |
| Anhui |  |  |
| … |  |  |
| Nationwide |  |  |

3.2 *Number of new diagnoses per year for NMOSD*

1. Search condition:
   1. Discharge diagnosis: C03C=NMOSD
   2. Geographical distribution: organization code (A01), birth address (A22), province of origin (A23C), and residence address (A24)
   3. Newly diagnosed cases are defined as patients who are first recorded in the HQMS database and primarily diagnosed with NMOSD.
2. Result output: Number of new diagnoses of NMOSD per year by province and country.

3.3 *Discharge department distribution of NMOSD*

1. Search condition:
   1. Discharge diagnosis: C03C or C06=NMOSD
   2. Discharge department: Discharge department (B16C)
2. Results output: Distribution of discharge departments of NMOSD from 2016 to 2018

3.4 *Age and gender distribution of patients with NMOSD*

1. Search condition:
   1. Discharge diagnosis: C03C or C06=NMOSD.
   2. Variables: age (A14), gender (A12C).
2. Results output: age and sex distribution of NMOSD from 2016 to 2018.

3.5 *Changes in medical care payment methods*

1. Search condition:
   1. Discharge diagnosisC03C or C06=NMOSD
   2. Variables: Medical payment method (A46C).
2. Results output: Changes in medical care payment methods of NMOSD from 2016 to 2018.

3.6 *Changes in hospitalization costs*

1. Search condition:
   1. Discharge diagnosis: C03C or C06=NMOSD
   2. Variables: hospitalization expenses including total hospitalization expenses, self-pay amount in total hospitalization expenses, nursing fees, imaging diagnosis fees, laboratory diagnosis fees, rehabilitation costs, western medicine costs, proprietary Chinese medicine costs.
2. Definition of annual hospitalization cost: number of patients per year= number of discharges-number of repeated discharges; annual average cost = annual total hospitalization cost / number of patients
3. Results output: Cost of hospitalization for NMOSD from 2016 to 2018

3.7 Change in length of hospitalization stay

1. Search condition:
   1. Discharge diagnosis: C03C or C06=NMOSD
   2. Variables: actual number of days in hospital (B20).
2. Definition of average length of stay. Number of Patients = Number of Discharges-Number of Repeat Discharges; Average Hospitalization Day = Length of Hospitalization / Number of Patient
3. Results output: length of hospitalization stay of NMOSD from 2016 to 2018.

3.8 Analysis of Co-existing disease

1. Search condition:
   1. Discharge diagnosis: C03C or C06=NMOSD
   2. Variables: Comorbidities (see in the below table).

| **Disease name** | **ICD-10 code** |
| --- | --- |
|  |  |
| Hypertension | I10, I12, I13 |
| Diabetes | E10, E11, I13, I14 |
| Osteoporosis | M80-M81 |
| Dyslipidemia | E78.0-E78.5 |
| Cerebral infarction | I63 |
| Intracerebral hemorrhage | I61 |
| Malignant tumor | C00-C97 |
| Arthritis | M05, M09 |
| Autoimmune disease | M30-M35 |
| Behcet's disease | M35.2 |
| Sjögren's syndrome | M35.0 |
| Systemic lupus erythematosus | M32 |
| Anxiety | F41 |
| Depression | F32 |

1. Results output: Comorbidities of NMOSD from 2016 to 2018 nationwide.

3.9 *Analysis of deaths*

1. Search condition:
   1. Discharge diagnosis: C03C or C06=NMOSD.
   2. Variables: Death (B34C); gender (A12C); age (A14); discharge department (B16C), cause of death.
2. Results output: Analysis deaths of National NMOSD from 2016 to 2018 nationwide.

**Reference**

1. Jiang L, Krumholz HM, Li X, Li J, Hu S. Achieving best outcomes for patients with cardiovascular disease in China by enhancing the quality of medical care and establishing a learning health-care system. *Lancet* 2015; **386**(10002): 1493-505.

2. Lin Y, Yang C, Chu H, et al. Association between the Charlson Comorbidity Index and the risk of 30-day unplanned readmission in patients receiving maintenance dialysis. *BMC nephrology* 2019; **20**(1): 363.

3. Han X, Zhou H. Monitoring traumatic brain injury in China. *The Lancet Neurology* 2019; **18**(9): 813.

4. Yang C, Wang H, Zhao X, et al. CKD in China: Evolving Spectrum and Public Health Implications. *American journal of kidney diseases : the official journal of the National Kidney Foundation* 2019.

| **Appendix** **Table 1 The number of tertiary hospitals involved in each province in this study.** | | | | |
| --- | --- | --- | --- | --- |
| **Province** | **Number of Hospital** | **Percentage（%）** | **Cumulative frequency** | **Cumulative percentage（%）** |
| Anhui | 45 | 2.7 | 45 | 2.7 |
| Beijing | 51 | 3.06 | 96 | 5.77 |
| Chongqing | 25 | 1.5 | 121 | 7.27 |
| Fujian | 47 | 2.82 | 168 | 10.09 |
| Gansu | 27 | 1.62 | 195 | 11.71 |
| Guangdong | 136 | 8.17 | 331 | 19.88 |
| Guangxi | 56 | 3.36 | 387 | 23.24 |
| Guizhou | 42 | 2.52 | 429 | 25.77 |
| Hainan | 18 | 1.08 | 447 | 26.85 |
| Hebei | 49 | 2.94 | 496 | 29.79 |
| Heilongjiang | 60 | 3.6 | 556 | 33.39 |
| Henan | 68 | 4.08 | 624 | 37.48 |
| Hubei | 74 | 4.44 | 698 | 41.92 |
| Hunan | 62 | 3.72 | 760 | 45.65 |
| Inner Mongolia | 44 | 2.64 | 804 | 48.29 |
| Jiangsu | 105 | 6.31 | 909 | 54.59 |
| Jiangxi | 51 | 3.06 | 960 | 57.66 |
| Jilin | 31 | 1.86 | 991 | 59.52 |
| Liaoning | 85 | 5.11 | 1076 | 64.62 |
| Ningxia | 9 | 0.54 | 1085 | 65.17 |
| Qinghai | 13 | 0.78 | 1098 | 65.95 |
| Shaanxi | 47 | 2.82 | 1145 | 68.77 |
| Shandong | 95 | 5.71 | 1240 | 74.47 |
| Shanghai | 35 | 2.1 | 1275 | 76.58 |
| Shanxi | 44 | 2.64 | 1319 | 79.22 |
| Sichuan | 144 | 8.65 | 1463 | 87.87 |
| Tianjin | 30 | 1.8 | 1493 | 89.67 |
| Tibet | 7 | 0.42 | 1500 | 90.09 |
| Xinjiang | 42 | 2.52 | 1542 | 92.61 |
| Yunnan | 32 | 1.92 | 1574 | 94.53 |
| Zhejiang | 91 | 5.47 | 1665 | 100 |

**Appendix Figure 1: The geographical distribution of 1665 tertiary hospitals in this study.** The study was conducted in mainland China. Hong Kong and Macao were not included.


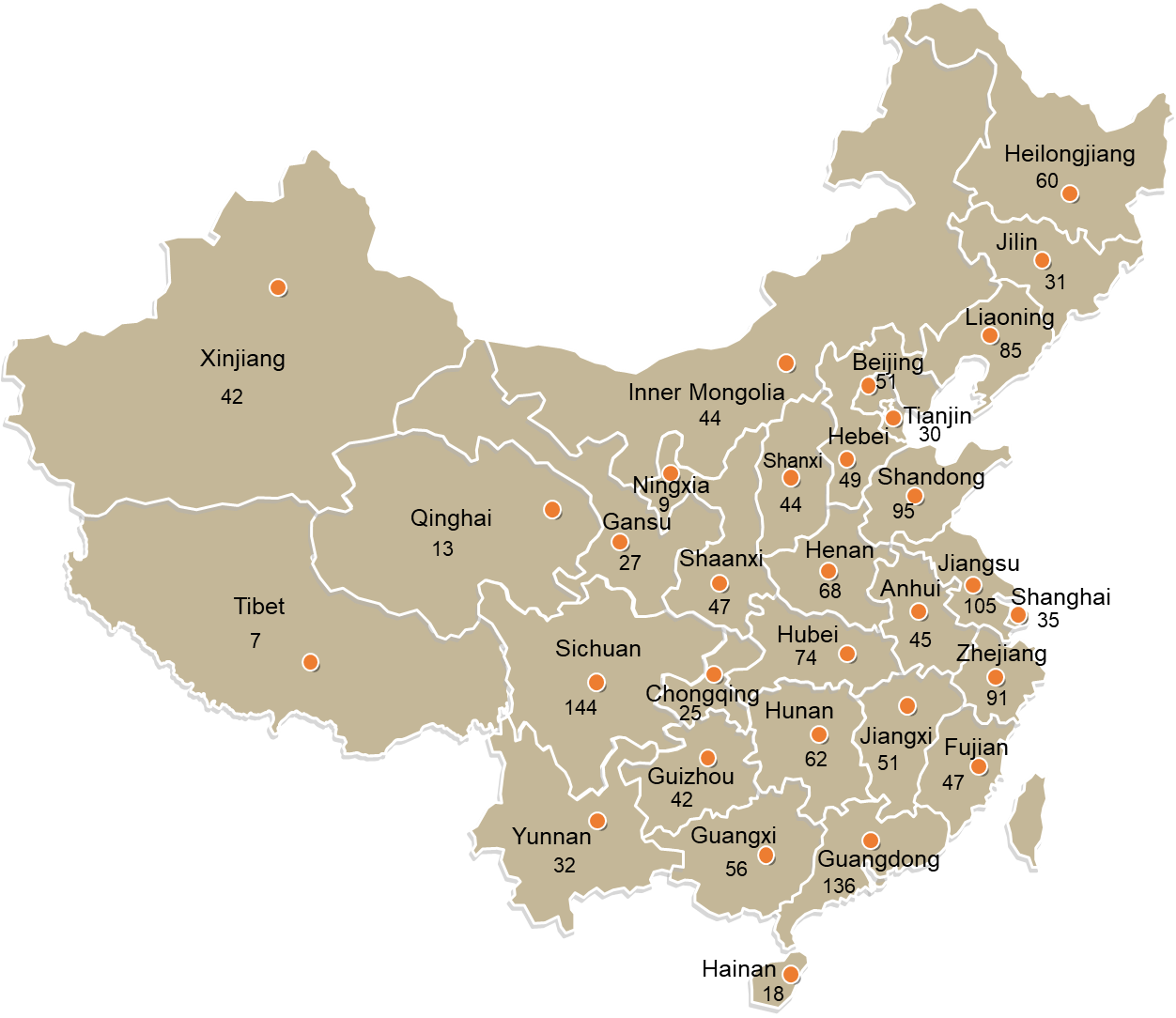

Supplement: Supplementary file 1 [file mmc1.docx]
