# Supplementary material for: Incidence of neuromyelitis optica spectrum disorder (NMOSD) in China: A national population-based study
Source: Lancet Reg Health West Pac. 2020 Sep 6;2:100021. doi: 10.1016/j.lanwpc.2020.100021 (PMC8315565; doi:10.1016/j.lanwpc.2020.100021)
Supplement: Supplementary file 3 [file mmc3.docx]

**中国视神经脊髓炎谱系疾病（NMOSD）的发病率：一项基于全国人群的研究**

田德财^1^，李子孝^1^，袁梦^1^，张程祎^1^，谷鸿秋^1^，王拥军^1^，施福东^1,2^

1.国家神经系统疾病临床研究中心，首都医科大学附属北京天坛医院，北京100070

2.天津医科大学总医院神经内科，天津市神经病学研究所，天津 300052

**背景** 视神经脊髓炎谱系疾病是星型胶质细胞被水通道蛋白4抗体攻击导致中枢神经系统炎性疾病，主要（较易）累及视神经和脊髓。尽管中国占世界人口的20%，涵盖了大部分的东亚人群，但中国NMOSD的发病率尚不清楚。

**方法** 我们基于中国医院质量监测系统（HQMS）的数据，完成了全国范围内的NMOSD发病率统计。该研究覆盖了中国大陆1665家三级医院。所有NMOSD患者的病案首页均依照统一标准进行收集。NMOSD的诊断标准依据2015年视神经脊髓炎谱系疾病诊断标准国际共识，ICD-10编码为G36·0。本研究的主要结果为NMOSD的发病率，同时，我们分析了NMOSD患者的住院负担、合并症及死亡情况。

**结果** 从2016到2018年，我们筛选出17,416名NMOSD患者的33,489份住院病历，其中11,973名患者是新发病例。年龄及性别调整后的NMOSD的发病率为0·278/10万人·年（95% [CI], 0.273-0·283），其中儿童为0·075（0·069-0·08）和成人为0·347（0·34-0·353）。发病的高峰年龄为45-65岁，发病率为0·445/100,000（95% CI, 0·433-0·457）。女性患者与男性患者的比例为4·71：1（p＜0·001, 95% CI, 4.50-4.94）。我们没有发现NMOSD发病率的地理分布与纬度有明确关系。NMOSD最常见的合并的自身免疫性疾病中为干燥综合征（1,124/17,416, 6·5%）和系统性红斑狼疮（387/17,416, 2·2%）。2016到2018年，在17,416名的NMOSD患者中，106名成人及4名儿童死亡。

**意义** 在中国，每10万人年的NMOSD发生率为0·278，其中儿童为0·075，成人为0·347。NMOSD发病率的地理分布与纬度梯度无关。

**基金**：国家自然科学基金（91949208, 91642205, 和81830038）;首都医科大学人脑保护高精尖中心

*Disclaimer: This translation in Chinese was submitted by the authors and we reproduce it as supplied. It has not been peer reviewed. Our editorial processes have only been applied to the original abstract in English, which should serve as reference for this manuscript.*
